# Seasonal home range dynamics and sex differences in habitat use in a threatened, coastal marsh bird

**DOI:** 10.1002/ece3.2761

**Published:** 2017-01-23

**Authors:** Jaan R. Kolts, Susan B. McRae

**Affiliations:** ^1^ Department of BiologyEast Carolina UniversityGreenvilleNCUSA; ^2^ Present address: USDA Forest ServiceRocky Mountain Research StationWildlife and Terrestrial Ecosystems Program322 East Front StreetSuite 401BoiseID83702USA

**Keywords:** coastal ecology, conservation, habitat preference, impoundment, radiotelemetry, Rallidae, *Rallus elegans*, seasonality, sexual segregation, wetland

## Abstract

A comprehensive understanding of spatiotemporal ecology is needed to develop conservation strategies for declining species. The king rail (*Rallus elegans*) is a secretive marsh bird whose range historically extended across the eastern United States. Inland migratory populations have been greatly reduced with most remaining populations inhabiting the coastal margins. Our objectives were to determine the migratory status of breeding king rails on the mid‐Atlantic coast and to characterize home range size, seasonal patterns of movement, and habitat use. Using radiotelemetry, we tracked individual king rails among seasons, and established that at least a segment of this breeding population is resident. Mean (±*SE*) home range size was 19.8 ± 5.0 ha (95% kernel density) or 2.5 ± 0.9 (50% kernel density). We detected seasonal variation and sex differences in home range size and habitat use. In the nonbreeding season, resident male home ranges coincided essentially with their breeding territories. Overwintering males were more likely than females to be found in natural emergent marsh with a greater area of open water. Females tended to have larger home ranges than males during the nonbreeding season. We report for the first time the use of wooded natural marsh by overwintering females. Brood‐rearing king rails led their young considerable distances away from their nests (average maximum distance: ~600 ± 200 m) and used both wooded natural and impounded marsh. King rails moved between natural marsh and managed impoundments during all life stages, but the proximity of these habitat types particularly benefitted brood‐rearing parents seeking foraging areas with shallower water in proximity to cover. Our results demonstrate the importance of interspersion of habitat types to support resident breeders. Summer draining of impounded wetlands that are seasonally flooded for wintering waterfowl allows regrowth of vegetation and provides additional habitat at a critical time for wading birds.

## 1 Introduction

Animal migration is an adaptive response to changes in ephemeral resources, intraspecific competition, and predator avoidance (Alerstam & Hedenstrom, 1998; Cox, 1968; Greenberg, 1980). Establishing migratory status is important for sustaining species at risk. Bird migration along a flyway introduces the possibility of gene flow among populations, whereas a resident, isolated population could have lower levels of genetic variability, potentially impacting population growth (Slatkin, 1987). If a declining species is migratory, preserving wintering or stopover habitat becomes of great importance. By contrast, the existence of residents is indicative of suitable breeding habitat that also meets the species’ needs year‐round.

An animal's home range can be influenced by density‐dependent variables such as population size (Wunderle, 1995), intraspecific competition (Greenberg, 1986), habitat quality (Kelley et al., 2011), and resource availability (Rolando, 1998). Food availability and spatial requirements may change seasonally resulting in modification of the home range (Takano & Haig, 2004). Habitat characteristics that affect home range size in turn impact local distribution and abundance (Morris, 1987). Moreover, individual variation in habitat use can lead to population structuring (Parrish & Sherry, 1994). Identifying critical habitat requirements for rare and declining species can inform the implementation and timing of management interventions. For instance, the intentional periodic flooding of impounded aquatic areas can enhance accessibility of vegetation and invertebrate species that benefit many avian wetland obligates (Conway, Eddleman, Anderson, & Hanebury, 1993).

Standardized marsh bird surveys have been effective in determining habitat occupancy and population trends (Budd & Krementz, 2011; Conway & Gibbs, 2005; Pierluissi & King, 2008). However, information on spatial requirements of rails (Family Rallidae) and other marsh birds remains sparse due to their secretive nature. The king rail *Rallus elegans* historically inhabited densely vegetated freshwater wetlands throughout the eastern United States. Alarming declines in abundance (Eddleman, Knopf, Meanley, Reid, & Zembal, 1988) have resulted in the king rail recently being uplisted globally to “Near Threatened” status (Birdlife International). In contrast to recent studies that have focused primarily on nesting habitat and site occupancy during the breeding season (Bolenbaugh, Cooper, Brady, Willard, & Krementz, 2012; Darrah & Krementz, 2009; Valente, King, & Wilson, 2011), our study investigated the intra‐ and interseasonal movements of king rails at one site on the Atlantic coast. Our approach was to examine habitat use of king rails on an annual cycle, as single‐season approaches cannot effectively identify an animal's spatial requirements to the extent necessary for developing conservation plans (Cline & Haig, 2011).

We used radiotelemetry to investigate seasonal movements, variation in home range size, and habitat use of the king rail at an island site on the mid‐Atlantic coast of the United States. Extensive freshwater to brackish marshes characterize this region of the North Carolina–Virginia coast, and Rogers, Collazo, and Drew (2013) found relatively high numbers of breeding king rails. Our first objective was to determine the migratory status of this coastal population. Although previously known to occur at this site both in winter and as breeders, it was not clear whether occupancy was by the same individuals. Migratory king rails breed to the north of this site in river basins flowing into the Chesapeake Bay (Meanley, 1969), but few are found there now (Timothy Freiday, pers. comm.). There is scant other information on residency patterns along the mid‐Atlantic coast in general. King and clapper rails (*Rallus crepitans*) have been studied in Beaufort County, South Carolina; although clapper rails were abundant and accessible, too few king rails could be captured for a radiotelemetry study (Ricketts, 2011).

Our second objective was to determine whether home range size varied between the breeding and nonbreeding seasons. Individual home range sizes of king rails were estimated in a study in coastal Louisiana (Pickens & King, 2013), but birds were tracked for 5 months or less during the breeding season. No previous study has characterized spatial requirements of king rails during the nonbreeding season. We predicted home ranges would expand during the nonbreeding season as resources became limited. We asked whether there were differences in movement patterns between the breeding and nonbreeding periods and between the sexes. We were particularly interested in movements and habitat use during the brood‐rearing period, about which little is known. Chicks remain flightless for 9 weeks and are vulnerable at this stage (Meanley, 1969). We interpret our results with respect to the implementation of management strategies that address species requirements at different life stages.

## 2 Materials and Methods

### 2.1 Study area

Research was conducted at Mackay Island National Wildlife Refuge (hereafter, “the refuge”) in the Intracoastal Waterway of northeastern North Carolina, between June 2012 and May 2014. This site was chosen for having the highest density of breeding king rails in the region (Rogers et al., 2013). The maintained refuge road system greatly facilitated access to the marsh for captures and tracking activities. The refuge's ~2,000 ha of freshwater natural marsh is subject to wind‐driven tides (Clauser & McRae, 2016b) and is dominated by emergent vegetation including black needlerush *Juncus roemerianus* (hereafter, *Juncus*), invasive common reed *Phragmites australis* (hereafter, *Phragmites*), and cattail *Typha sp*. Other habitats include mixed hardwoods and pinewoods. The refuge also has over 350 ha of impoundments managed via water control structures for migrating and wintering waterfowl and other wetland birds.

### 2.2 Capture methods, morphometrics, and transmitter design

Methods used to capture king rails varied by season. We conducted spotlighting from an airboat at night in the nonbreeding season. During the mate‐finding period, we deployed a whoosh net with a call lure. To catch breeders, we used mist nets to surround their nests during the last week of incubation (for additional details, see Clauser & McRae, 2016a). Each adult was banded with a U.S. Geological Survey numbered aluminum band and a unique combination of three colored spiral darvic flat bands (A.C. Hughes, UK), distributed as two on each leg. On application, darvic bands were sealed in a ring with a cordless welding iron.

Basic morphometric measurements were taken on captured adults: bill length (±0.1 mm) and tarsus length (±1 mm) were measured with dial calipers, “tarsus and middle toe” and flattened wing chord (±1 mm) were measured with a 300‐mm wing rule, and body weight (±5 g) was measured with a 500‐g Pesola. Adults meeting minimum body size requirement (weighing at least 320 g) were fitted with a radio transmitter (Advanced Telemetry Systems, Isanti, MN; Model A2480, 3.6 g) with a backpack harness made of Teflon ribbon (Bally Ribbon Mills) adapted from the design of Dwyer (1972) (total weight ~9 g; see also Casazza, Overton, Takekawa, Rohmer, & Navarre, 2008). The transmitter had an estimated battery life of 8.5 months. The transmitter and harness assembly was ≤3% (range: 1.8–2.9%) of the bird's total body weight.

### 2.3 Sex diagnosis

At the time of banding, a 50‐μL blood sample was drawn from the brachial vein and stored in ~1.5 mL 100% ethanol. Sex could often be determined from morphometrics with males being larger on average than females in all measures. However, size distributions for males and females overlap, so we used DNA extracted from the blood samples to conduct a polymerase chain reaction (PCR)‐based diagnostic test. We amplified a segment of an intron of the CHD (chromo‐helicase DNA‐binding) gene (Perkins, King, Travis, & Linscombe, 2009), but we used as a downstream primer, 1237L from Kahn, St. John, and Quinn (1998). DNA was extracted using the “animal tissue protocol” of the DNeasy Tissue Kit (Qiagen Inc.). A small amount (~2 mm^2^) of blood cells was briefly air‐dried before overnight digestion with 0.4 mg Proteinase K in buffer at 55°C. Following extraction and washes, DNA was stored in elution buffer at −20°C. Each 10‐μL reaction contained ~ 50 ng genomic DNA, 5 pmol of each primer, P2 (fluorescently labeled with 6‐FAM, Bioneer) and 1237L, 0.2 mM dNTPs, 2.5 mM MgCl_2_, and 0.5 Units Taq (recombinant) polymerase (Invitrogen or Bioline). PCR was performed in a PTC‐100 thermocycler (MJ Research) starting with an initial denaturation step at 94°C for 120 s, and cycling 30 times through 94°C for 30 s, 50°C for 45 s, and 72°C for 60 s, and a final 5‐min 72°C extension step. PCR products were visualized on an ABI 3130xl Genetic Analyzer. Alleles were sized in relation to GeneScan 600 LIZ size standard (Applied Biosystems) using GeneMapper software (version 4.0; Applied Biosystems).

### 2.4 Field protocol and spatial analysis

All fixes from radio‐transmitters were manually acquired using a portable handheld receiver (Advanced Telemetry Systems, model R410) and a three‐element folding Yagi antenna. We typically tracked individuals within a few hours to determine whether they were moving, but the first location with habitat data was recorded at least 24 hr after attachment of the transmitter, in case capture may have influenced their movements. Thereafter, locations of each individual were documented at least 20 hr apart to ensure independence. King rails with transmitters were tracked every 1–3 days during the breeding season (April 1‐August 31). Intervals between fixes during this period were determined based on tracking schedules of concurrently tagged individuals and the distances between them, as well as other fieldwork priorities and weather. For concurrently tagged individuals, the order of tracking was varied, determined by the need to economize travel time and distance to complete other essential fieldwork activities including catching birds and nest monitoring. During the nonbreeding season, king rails were tracked at least twice every 2 weeks (September 1‐March 31). Teaching obligations of the authors prevented us from being able to track birds more frequently during those months. Takano and Haig (2004) used a similar hybrid schedule to track rails year‐round.

Individuals were tracked at varying times between sunrise and sunset (usually between 06:00 and 18:00 h Eastern Summer Time during the breeding season). Scheduling was opportunistic as there was often a need to track two or more individuals in distal parts of the refuge. This was balanced with the need to complete other time‐sensitive field activities such as captures and nest monitoring. On some occasions during the nonbreeding season and early breeding season, it took hours to locate a bird. This varied according to habitat type (signals tended to be weaker and more attenuated in wooded areas) and how far the birds had moved.

Fixes were obtained in one of two ways. First, we triangulated bird locations by taking compass bearings from three to five preset locations (hereafter, “tracking stations”). All bearings on an individual were gathered within 30 min of one another to minimize error caused by large movements. Triangulation locations were digitized using the maximum likelihood estimation method as computed by LOAS software (Location Of A Signal, version 4.0; Ecological Software Solutions LLC). Ellipse errors were calculated from the data using a 95% confidence interval with a χ^2^ distribution. We eliminated 15 locations (3%) with an error ellipse >10 ha from three different birds because the birds had likely moved during triangulation.

Second, we conducted “walk‐ins” where birds were followed in the marsh until visual or auditory detection was made. These were necessary to characterize specific microhabitat use. Birds caught while incubating were monitored more frequently for possible changes in behavior or location directly before and after their clutches hatched. Locations of brood‐rearing adults were also documented every 1–3 days. Brood success was measured based on visual or auditory confirmation of chick presence.

During “walk‐ins,” the following microhabitat characteristics were documented by visual estimation within a 10‐meter radius of the bird location, and at a “random” location determined by spinning an analog compass to select an azimuth and walking 50 m from the bird location (following the method of Pickens & King, 2013) as determined by handheld GPS (Garmin): percent cover of open water and each plant species >5%, percent woody canopy (woody species >3 m in height), and distance to open water and edge (defined as any ecotone or transition in canopy cover or major dominant reed species, e.g., *Juncus/Phragmites*). We estimated that the minimum breeding territory size during nesting was about 1 ha, so these points would reasonably represent available habitat within the pair's territory where they might alternatively have nested, while being as far as possible without the risk of being outside of territory range. Open water was defined as at least one square meter of water (as in Pickens & King, 2013). This was considered suitable as a king rail foraging patch in our study based on frequent observation of crayfish shell debris together with king rail footprints in mud next to pools of this size or larger. Water depth of pools was also measured with a ruler (±1 cm). The distance to open water was also inclusive of larger ponds, water channels, or impoundments.

Using the computational options of the BIOTAS software (version 2.0; Ecological Software Solutions LLC), home ranges were calculated under a fixed kernel density analysis with least squares cross‐validation as a smoothing parameter. In an effort to include data from as many of our radio‐tagged birds as possible, while still maintaining the integrity of the fixed kernel density analysis, we estimated home ranges for king rails with ≥14 point locations. That is, in cases where we quantified home ranges from breeding and nonbreeding periods separately, each had a minimum of 14 points. Although small numbers of observations tend to overestimate home range size (Seaman et al., 1999), Börger et al. (2006) demonstrated that for roe deer (*Capreolus capreolus*) and common kestrel (*Falco tinnunculus*), home ranges normalize after collecting 10 fixes. In coastal Louisiana, king rail home range sizes typically did not increase after 20 fixes (Pickens & King, 2013).

To determine whether home range size differed between the breeding and nonbreeding periods, we used a paired sample t‐test for the subset of birds for which sufficient data were collected during both time periods. We combined the variables from bird point locations and random locations to describe microhabitat variables within a home range, because only six of the 191 (3%) random points fell outside of the 95% kernel density estimate contour for all birds sampled. We ran Pearson's correlations to detect habitat associations with home range size for 95% and 50% kernel densities. We tested for effects of sample size on home range size by running a paired samples t‐test on home ranges with >40 points and randomly subsampling the data points to 30 and to 20. We also tested for an effect of tracking duration (the amount of time in days that an individual was followed) on home range size with a linear regression model. We calculated median, mean, and maximum distance between locations using Geospatial Modelling Environment (GME version 0.7.2.1, Spatial Ecology LLC) for each bird. To compare differences in movement patterns between sexes, we used an independent samples t‐test. We calculated separately movement rates of adults with broods. Results are reported as means ±*SE* and figure error bars are ±1 *SE*.

## 3 Results

### 3.1 King rail radiotelemetry

**Figure 1:**
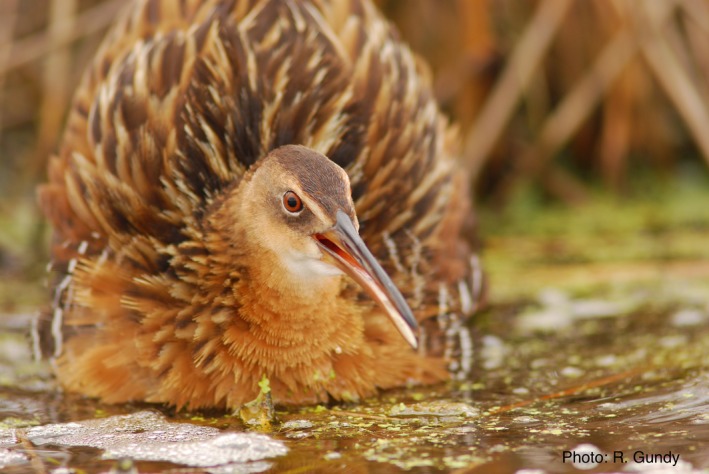
An adult king rail bathing near its nest (Photograph: Robert Gundy)

**Figure 2:**
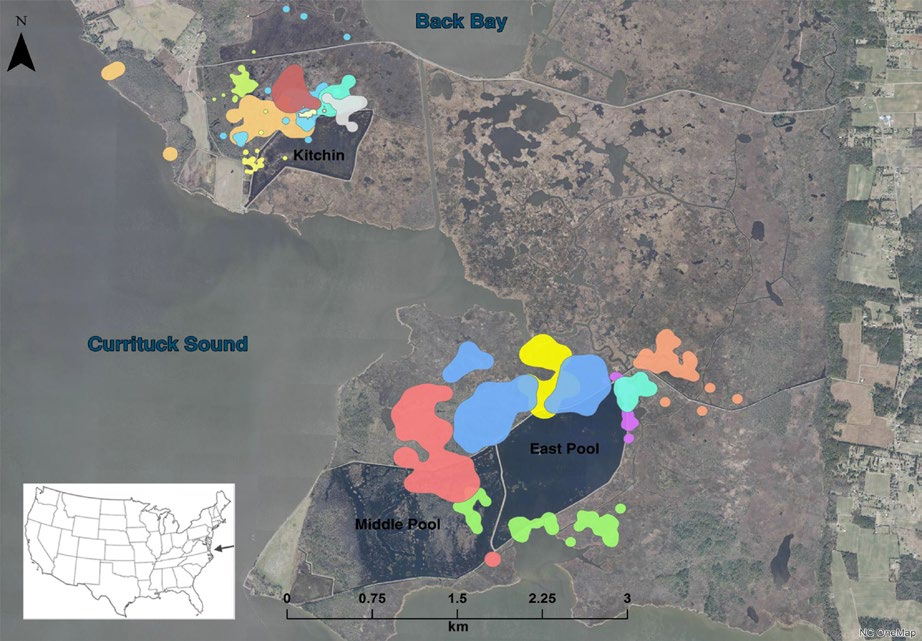
Home ranges of king rails based on radiotelemetry. Each color represents a single individual's home range (*N *=* *15), but the tracking time frames do not necessarily overlap. Individuals did not move between northern and southern sections of the refuge. Dark areas of map are open water impoundments labeled Kitchin, East Pool, and Middle Pool. Pale lines are roads. Currituck Sound and Back Bay are interconnected and form part of the Intracoastal waterway that is separated from the Atlantic Ocean by a narrow land barrier that constitutes the northern extent of North Carolina's Outer Banks and Virginia's tidewater region on the east coast of the USA (inset)

A total of 21 king rails (Figure 1) were captured and radio‐tagged between 10 June 2012 and 11 March 2014. Six were captured in the nonbreeding season by spotlighting at night from an airboat, and 15 in the breeding season: three using a whoosh net early in the breeding season (April) and 12 on the nest late in incubation. Of 576 bird locations, 145 were walk‐ins (25%). We estimated home ranges for 15 king rails (*n* = six females and nine males) with ≥14 data points (median = 42 per bird, range = 14–69) between 15 June 2012 and 28 January 2014 (see Figure 2 for a map of all king rail home ranges). The mean bearing error of telemetry signals from tracking stations was 2.68 ± 1.14°.

### 3.2 Seasonal and sex‐related variation in home range size

**Figure 3:**
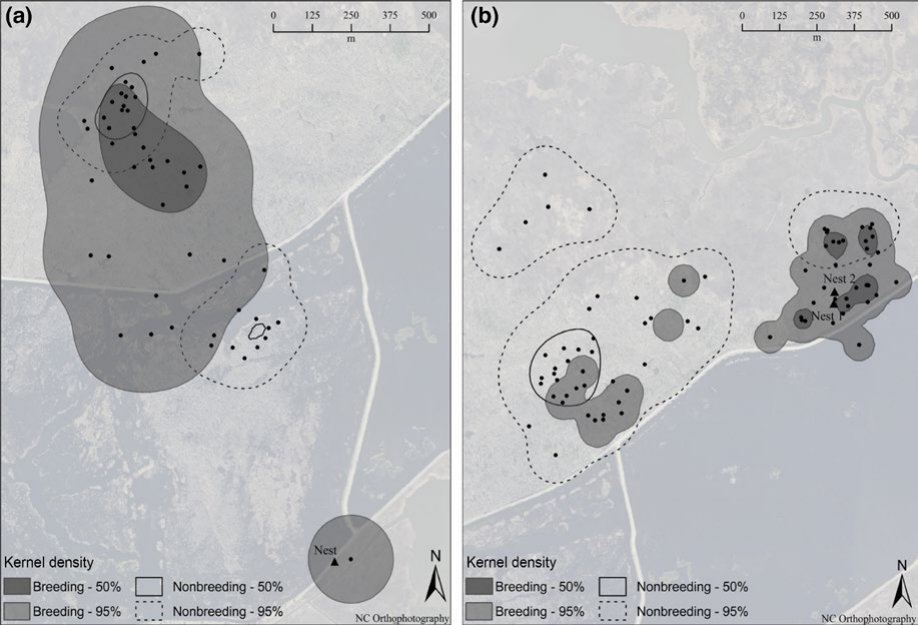
Variation in female King Rail home ranges between breeding and nonbreeding seasons. Examples shown are for two females (a and b) each caught on their nests. Points represent bird locations and nest locations are labeled. In b, the female was caught on Nest 1, which failed, but her renest, Nest 2, was successful. Both females brought their broods to different parts of the same wooded marsh (top left in a, mid‐left in b). Both overwintered in this habitat. Female a led her brood 1,008 m north from the nest only 5 days after the last chick fledged (see text)

**Figure 4:**
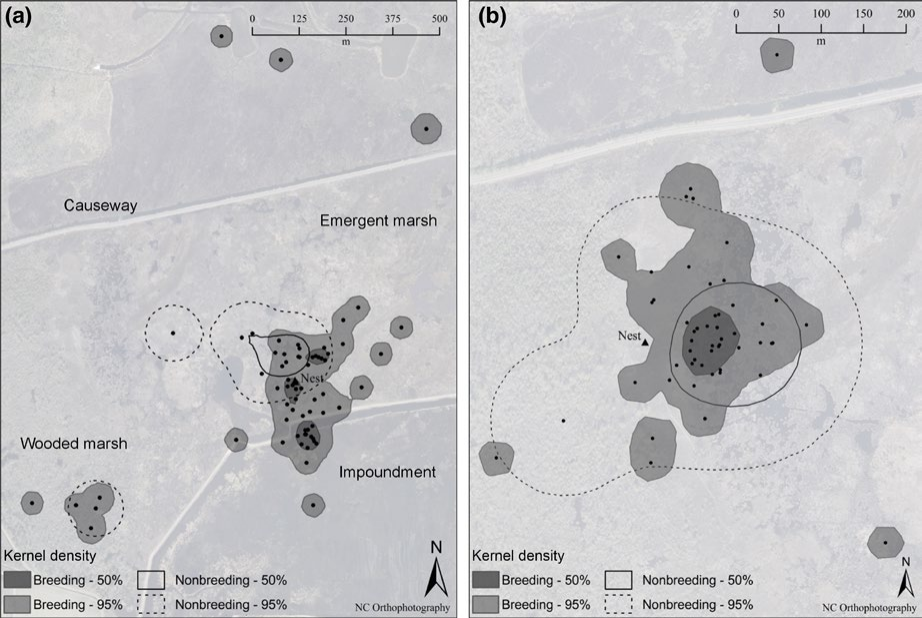
Variation in male King Rail home ranges between breeding and nonbreeding seasons. Examples shown are for two males (a and b) both caught via spotlighting from an airboat, and followed as they paired, nested, and tended broods. Points represent bird locations, and nest locations are labeled. Outlying points represent forays by each male during the premating period, during which time they advertised for a mate using “kek” calls. For example, Male a forayed 600 m west into wooded marsh for the first 2 weeks of April before returning to the center of his territory. One week later, Male A then forayed 900 m to the north in emergent marsh across the causeway for 4 days. During these forays, Male a engaged in bouts of “kek” mating calls. He returned to his territory with a mate

Contrary to expectation, home ranges tended to be larger during the breeding season than during the nonbreeding season. However, as nine of the 13 birds for which we obtained breeding season data were captured late in incubation, many breeding season points were gathered during the brood‐rearing period (*n *=* *95) when parents are no longer constrained to return to their nests. Considering only individuals adequately sampled during both periods (*n *= three males and five females; e.g., Figures 3 and 4), there was no significant difference in home range size between breeding and nonbreeding periods (paired samples, 95% kernel densities, *t*_7_ = 1.12, *p *=* *.30; Table 1). Nevertheless, visual inspection revealed that for males, there was substantial overlap in the core area in which the nest was situated. By contrast, the core area shifted away from the nest site and was situated in wooded marsh for four of the five females during the nonbreeding season (e.g., Figure 3b).

**Table 1:** Among‐season differences in king rail home range sizes. Values presented are means ± *SE* with ranges in brackets

Season	Number of birds tracked	Mean (range) independent locations	95% kernel density (ha)	50% kernel density (ha)
Breeding (Apr 1 ‐ Aug 31)	13	28 (15–48)	22.5 ± 6.9 (1.0–70.8)	3.1 ± 1.1 (0.07–13.3)
Nonbreeding (Sept 1 ‐ Mar 31)	10	21 (14–34)	15.6 ± 3.5 (0.5–34.4)	2.1 ± 0.5 (0.07–4.9)
Annual average	15	38 (14–69)	19.8 ± 5.0 (1.0–69.8)	2.5 ± 0.9 (0.1–13.6)

To investigate whether there was an effect of sample size (number of fixes) on home range size, we compared home range sizes of seven birds with >40 data points each (range: 42–69) with their estimated home range sizes after randomly subsampling 30 points each (paired samples *t*_6_ = −1.27, *p *=* *.25) and 20 points each (paired samples *t*_6_ = −1.85, *p *=* *.11). Thus, randomly reducing the number of data points did not significantly affect the measure of home range size. Notwithstanding, the total number of days that individuals were tracked explained a significant proportion of variance in home range size (*R* ^2^ = .48, *F*_1,13_ = 11.4, *p *=* *.006). However, this was strongly influenced by data from one female that was tracked for 368 days, 120 days longer than the next longest tracking duration. When this female's data were removed from the analysis, tracking duration was not a significant factor in the model (*R* ^2^ = .19, *F*_1,12_ = 2.6, *p *=* *.13).

Including four captures from earlier years in the study, nine of 10 king rails captured between November 3 and March 9 were male. Captures in winter were all in open marsh habitat that was navigable by airboat, supporting differences in habitat use between the sexes during the nonbreeding season. We found that during the nonbreeding period, males had significantly smaller 95% kernel density home ranges (8.3 ± 2.8 ha) than females (23.0 ± 4.9 ha; *t*_7_ = −2.40, *p *=* *.047).

Most microhabitat variables measured in this study did not differ between the sexes. However, males had a significantly greater percentage of open water within their home ranges (34 ± 3%) than females (24 ± 3%; *t*_11_ = 2.25, *p *=* *.046). Female home ranges contained a significantly greater percentage of southern wax myrtle *Myrica cerifera* (18 ± 1%), a shrub species found in the understory of wooded wetlands, than male home ranges (9 ± 3%; *t*_5_ = 2.85, *p *=* *.036).

### 3.3 Individual movements

The overall mean distance traveled between locations was 262 ± 44 m, and the average maximum distance per individual was 884 ± 169 m (*N* = 15 birds). Mean distance moved between fixes did not differ significantly between the sexes (means = 347 ± 65 m (six females), 205 ± 53 m (eight males), *F*_1,13_ = 2.87, *p *=* *.11), nor did maximum distance travelled (mean maximum = 1,131 ± 263 m for 6 females, 718 ± 215 m for 8 males, *F*_1,13_ = 1.48, *p *=* *.25).

Of eight king rails tracked at the refuge during both the breeding and the nonbreeding seasons (three males and five females), seven remained on the island, confirming year‐round residency of these individuals. The exception was a breeding female, captured on 27 June 2013 on the nest, and documented on the island for 215 days. Her signal was lost between 3 August and 17 September 2013, but was detected again after 17 September 2013, in wooded marsh habitat 450 m from her nesting location. She was last detected on 28 January 2014, moving west across a ~5‐km expanse of the Intracoastal Waterway toward the mainland. The transmitter battery would most likely have died before her next breeding attempt, but her nonbreeding home range was likely greatly underestimated. Another female captured on 23 June 2012 moved 418 m on 8 August 2012, from her nesting area to a wooded marsh. She remained within the wooded marsh for 248 days before moving back to her previous nesting area in April, 2013.

Only one male king rail (of nine) was tracked in wooded marsh habitat during the study period. This male was radio‐tagged on 2 March 2013. On March 31, he moved 600 m from his territory and remained in the wooded marsh until April 13. During this time, he was giving “kek” calls that are known to function in mate attraction (Zembal & Massey, 1985). He briefly returned to his territory and then moved 900 m north of a major road, “Causeway”, that bisects the northern extremity of the marsh. He returned to his territory with a mate 4 days later (Figure 4a). These were the farthest forays by a male away from his core breeding territory, but we also documented mate‐searching forays by other males (e.g., Figure 4b).

Anecdotal records from additional king rails that were not radio‐tagged support that this breeding population is at least partially resident. One male captured while spotlighting from the airboat in February 2013 was found incubating a clutch the following June. Through DNA analysis, a male captured during the winter of 2012 was retroactively determined to be the father of a brood from 2011 (C.L. Brackett and S.B. McRae, unpublished).

### 3.4 Brood movements

We tracked six king rail parents (four males and two females) during the brood‐rearing period. As broods became more mobile 2 weeks posthatching, they became more difficult to detect in the emergent vegetation, often stealthily moving away with the adult. The longest period of time a brood was confirmed to be with the radio‐tagged parent was 39 days posthatching (Hatch day = Day 0).

In the first 2 days after fledging, broods moved a mean distance of 89 ± 27 m from their nests. Between 3 and 5 days posthatching, broods moved a mean distance of 312 ± 144 m from their nest sites. Broods moved on average 157 ± 13 m between sightings (an approximation of their mean daily movement rate). Over the course of the dependent period, broods moved a mean maximum distance of 581 ± 211 m from their nests over a mean of 28.0 ± 4.5 days (range = 8–39). The greatest distance traveled by a brood was more than one kilometer in 1 day. The radio‐tagged female led her brood from the vicinity of the nest site in emergent marsh, across an impoundment to a wooded marsh 5 days after the last chicks hatched (Figure 3a). The family may have alternatively travelled along a refuge road with little to no cover. However, this brood was subsequently led from the natural wooded marsh back into the impoundment when a 5‐day high‐water event caused by strong sustained south winds occurred in the natural marsh.

**Figure 5:**
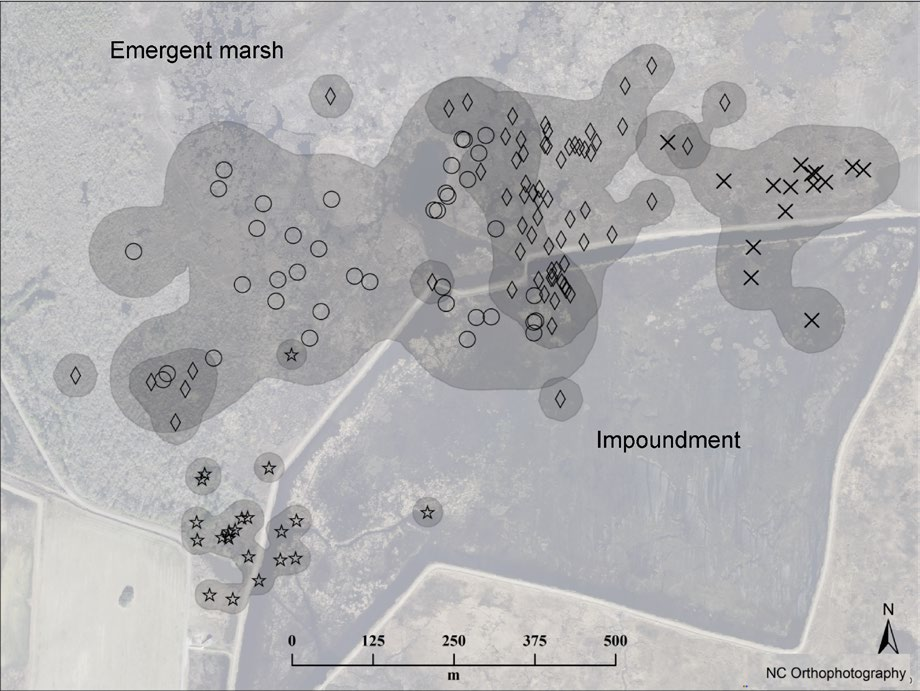
Depiction of four king rail home ranges incorporating the Kitchin impoundment during the 2013 breeding season. Each individual's locations are marked with a different symbol. The overlapping cluster of diamonds and circles depicts locations recorded between 20 June and 26 July 2013, after two broods from adjacent territories hatched (on 20 June and 28 June, respectively), and were being tended in proximity to one another. In both cases, brood‐rearing parents moved between natural marsh and managed impoundments

Three of six radio‐tagged brood‐rearing parents fed their broods in impoundments. Although the unbanded (nontracked) parent was rarely visible, dual alarm calling was commonly heard during walk‐ins confirming the presence of two parents with the brood. Two broods that hatched synchronously with one another in adjacent territories were regularly observed moving between emergent natural marsh and impounded marsh (Figure 5). The adults foraged in the impoundment and carried crayfish to their broods waiting in patches of cattail *Typha* that are broader leaved than the predominant black needlerush *Juncus* providing better canopy cover. Cattail stems are softer and grow at lower densities that may be easier for chicks to navigate. A third brood hatched from a nest immediately adjacent to an impoundment where the parent was observed foraging on five separate occasions. The brood was brought to an area dominated by invasive *Phragmites* with small patches of native marsh vegetation, and remained in that area for 5 weeks, after which time we could no longer detect the chicks. We observed three other broods tended by non‐radio‐tagged parents using impoundments, including a color‐banded adult with 3‐week‐old chicks.

### 3.5 Impoundment use by king rails

Impoundments on the refuge are routinely drawn down in spring, after what is estimated will be the last cold front of the spring season carrying a strong north wind. The natural marshes at the refuge are subject to wind‐driven tides (Clauser & McRae, 2016b), and north winds lower the water levels allowing the necessary negative pressure to drain the impoundments in 3–4 days. Drawdown is typically conducted between mid‐April and late May. On 14 May 2013, between 0.6 and 0.8 m of the water depth was drained from the impoundments, initially creating expansive mudflats with sporadic *Phragmites* and *Typha sp*. cover. These gradually became overgrown over the course of the summer with vegetation beneficial for wintering waterfowl, including submerged aquatic vegetation, emersed aquatic vegetation, and moist soil plants, such as millet *Panicum miliaceum*, wild rice *Zizania sp*., beggarticks *Bidens sp.,* and Eurasian milfoil *Myriophyllum spicatum*. Prior to the day of drawdown, radio‐tagged king rails had been tracked only in natural marsh. One male, radio‐tagged in March, was detected in an impoundment for the first time on the day of drawdown. Thereafter, 15 of 68 points (22%) comprising his home range were within the impoundment where he was observed foraging. Three additional radio‐tagged king rails frequented the same impoundment during the breeding season after drawdown had occurred (Figure 5).

Two king rails were tracked within a pair of adjacent impoundments (East Pool and Middle Pool, Figure 2) during the breeding and nonbreeding seasons with a total of 21 of 40 locations (52.5%) and 15 of 50 locations (30%). An additional radio‐tagged female was located in an impoundment for 2 days between her nesting attempts in natural marsh. Three nesting attempts were initiated in an impoundment in early June, although none were successful. Their fates were unrelated to water management: Each was depredated. Additional king rail detections were made by way of vocalizations: These were commonly heard in impoundments.

All king rails with home ranges adjacent to impoundments foraged both in the impoundments and in natural marsh, except during the nonbreeding period when water levels were high. We predicted that adults frequenting impoundments would have smaller home ranges, because they may not have had to travel as far between prey items when foraging. However, home ranges adjacent to impoundments were, on average, not different in size from home ranges that were not (*n *=* *7 adjacent and 8 nonadjacent; *t*_14_ = 0.05, *p *=* *.96). There was a negative correlation between breeding home range size and percent open water (Pearson's correlation, 95% kernel: *R *=* *−.62, *n *=* *11, *p *=* *.04), but no correlation during the nonbreeding period.

## 4 Discussion

Tracking individual king rails throughout the year at a site along the Atlantic coast provided insights into the spatiotemporal ecology of a declining and secretive marsh bird. We determined that some individual king rails maintain year‐round residency. The presence of radio‐tagged individuals during both breeding and nonbreeding seasons, as well as anecdotal evidence from a small number of additional individuals, supports that at least a segment of the population is resident. Uncertainties still remain regarding the migratory status of populations along the mid‐Atlantic coast (Meanley, 1969). The possibility that there is admixture of migrant and resident populations at this site awaits genetic confirmation (Brackett, Maley, Brumfield, & McRae, 2013). The combination of natural marsh and managed impoundments at this refuge, under current management practices, is conducive to supporting a relatively large breeding population year‐round. Identifying wetlands with similar habitat characteristics along the Atlantic coast and introducing similar management practices could assist species recovery efforts.

The estimated mean breeding home range sizes of king rails in this study were larger than those reported in Louisiana (0.8–32.8 ha) where adults were tracked for no more than 5 months during the breeding season (Pickens & King, 2013). Differences could have been due in part to regional habitat differences, food availability, or possibly higher population density in Louisiana. Yet, mean daily movement distances between sightings were similar on average to radiotelemetry studies of Gulf Coast king rails (78–144 m) (Pickens & King, 2013), as well as to those of Ridgway's rails *Rallus obsoletus yumanensis* (126–157 m) (formerly Yuma clapper rail; Conway et al., 1993). The larger home ranges reported in this study are more likely due to following individuals for longer time periods, on average. This gives a more accurate representation of the spatial requirements of resident individuals during the annual cycle.

Tracking in this study was conducted with a conventional handheld receiver, and we were not able to be out in the field as often in the nonbreeding season compared to the breeding season. In a study of Mariana moorhens *Gallinula chloropus guami*, sampling during the rainy (nonbreeding) season occurred at a reduced frequency, likely for similar reasons (Takano & Haig, 2004). Taking points less frequently during the nonbreeding season may have actually reduced the possibility that we inadvertently altered the movements of rails at a time when birds may feel more vulnerable and flighty. New satellite and remote stationary receiver technologies will eliminate these concerns in future studies.

Importantly, the inclusion of location data from brood‐rearing adults in this study raised the average breeding period home range size; young families ranged over unexpectedly long distances. As territories broke down and parents led their broods among habitats, they greatly expanded their home ranges. Our study highlights the importance of investigating year‐round space use and habitat requirements during different life stages.

Although within‐individual comparisons were equivocal, contrary to our predictions, king rail home ranges decreased in size, on average, during the nonbreeding period. We had predicted that home ranges would increase during the winter due to a decrease in food availability. The king rail diet is known to vary considerably throughout the year, with animal prey constituting nearly 90% of its spring and summer diet but only 58% of the winter diet (Meanley, 1953). Anecdotally, we observed that there was still abundant terrestrial arthropod prey in the marsh through October when other migratory rails arrive from northern breeding grounds. A decrease in invertebrate abundance during the winter may compel king rails to revert to using smaller areas with a greater abundance of edible plants such as browntop millet (*Panicum ramosum*) found within impoundments, tubers of arrowhead (*Sagittaria*), and woody plant seeds (Meanley, 1953; Nassar, Chabreck, & Hayden, 1988). *Carex* is found in wooded marsh at the study site and is an important food source for the yellow rail (*Coturnicops noveboracensis*) (Robert, Cloutier, & Laporte, 1997). Seeds constitute 98% of the diet of migrating soras (*Porzana carolina*) in freshwater marshes (Webster, 1964).

During the nonbreeding period, rails rarely vocalize and sightings are uncommon (Conway et al., 1993; Meanley, 1969), so it is conceivable that an undetected influx of overwintering king rails arriving from more northerly breeding populations could cause a contraction in winter home ranges. This could be influenced further by dynamic coastal processes: Coastal freshwater marshes in this region are subject to wind‐driven tides. In winter months, strong sustained north winds decrease water levels to reveal expansive mudflats that may temporarily increase foraging opportunities leading to home range contraction.

Male king rails had smaller home ranges on average than females during the nonbreeding period. Males’ home ranges also had a greater percentage by area of open water, on average, suggesting that males remain in higher quality habitat. The extent of shallow water is expected to be associated with food as king rails feed primarily on benthic macroinvertebrates, particularly crayfish (Meanley, 1969). In saltmarshes along the Gulf Coast, clapper rail home ranges varied in size with smaller home ranges being associated with greater densities of fiddler crabs *Uca sp*. (Rush, Mordecai, Woodrey, & Cooper, 2012). In the brackish marshes of coastal Louisiana, home range size was inversely related to percent cover of open water; king rails in locations with more open water also had smaller core areas and tended to have shorter maximum displacements between fixes (Pickens & King, 2013).

Recent surveys have reported that king rail occupancy was inversely related to the presence of woody cover (Darrah & Krementz, 2009; Pierluissi & King, 2008). However, these studies were conducted exclusively in the breeding season. Meanley (1969) observed that king rails in central Louisiana could be found overwintering in longleaf pine *Pinus palustris* woodlands endowed with damp hollows for crayfish, and also among stands of loblolly pine *Pinus taeda* in Maryland wetlands with a thick year‐round ground cover of switchgrass *Panicum virgatum*. Remarkably, in our study, the majority of king rails captured overwintering in the open marsh were male. Whereas four of five females tracked during this period resided in wooded marsh with stands of loblolly pine, suggesting sexual segregation in habitat use during the winter, only two males were observed in wooded marsh during the study. One entered a wooded area with its mate and brood and was depredated 2 days later. Another moved into the woods for 3 days in the spring, notably while advertising for a mate.

Female home ranges had a greater percentage of southern wax myrtle, a common shrub species offering canopy cover and potentially also forage. Clapper rails wintering along the Atlantic coast have been similarly observed to move from low to high marsh with a greater abundance of southern wax myrtle (Adams & Quay, 1958). Females tended to move greater distances between successive tracking points than males during both the breeding and the nonbreeding periods, suggesting that females may be using marginal habitat, compelling them to travel farther to forage. We were unable to follow one female breeder after she appeared to have left the island in the middle of the nonbreeding period. Thus, we likely underestimated the mean female nonbreeding home range size. This also raises the possibility of a partial migrant breeding population, possibly segregated according to sex.

Sexual segregation in habitat use has never before been described in rails, but it is well documented in various Neotropical migrants (Morton, 1990; Ornat & Greenberg, 1990; Parrish & Sherry, 1994). For example, males have been shown to defend higher quality habitat on wintering grounds, while females use marginal habitat (Parrish & Sherry, 1994; Wunderle, 1995), presumably due to competitive exclusion by males, with food availability likely being the driving force behind habitat selection during the winter period (Parrish & Sherry, 1994). Resident male king rails remained on their breeding territories during the winter, and likely benefited from both familiarity and greater food availability following the departure of their mates. Similar to the Townsend's solitaire *Myadestes townsendi* (Salomonson & Balda, 1977), as pair bonds break down after the breeding season and resources decrease in winter, females may remain in the area as nonterritorial floaters. Whether or not female use of wooded wetlands has adaptive value or occurs out of constraint remains to be determined.

King rail broods moved a substantial distance from their nests within the first few days of hatching. Considering chicks weigh an average of only 14–16 g when they hatch (J. Kolts and S. McRae, unpublished data), movements of 100 m over variable terrain is remarkable. As in mallard *Anas platyrhynchos* broods (Mauser, Jarvis, & Gilmer, 1994), most relocations occurred within the first week as king rail broods became progressively more mobile between 3 and 5 days posthatching. However, in contrast to the precocial chicks of waterfowl, king rail chicks are dependent on parental feedings (Meanley, 1969). King rail broods were never in the same location between successive tracking days. Frequent moving may indicate prey depletion (Brinkhof, 1997), or they may move in relation to variation in water level which can limit prey accessibility and offspring mobility (Bancroft, Gawlik, & Rutchey, 2002). Chicks of willow grouse *Lagopus lagopus* move more frequently in areas with lower insect abundance, and adults with broods traveled greater distances than those without to satisfy the nutritional needs of their young (Erikstad, 1985). A family of king rails could quickly deplete an area of accessible invertebrate prey.

The increased movements of brood‐rearing parents may alternatively reflect predator avoidance. Estimated survival rates in the Midwest and Gulf Coast regions are between 4–15% for king rail broods within the first 2 weeks of hatching (Darrah & Krementz, 2009; Pickens & King, 2013). Survivorship estimates could not be made in this study due to the difficulty in visual confirmation of the number of surviving chicks. Persistent alarm calling from attending parents frustrated attempts at auditory confirmation. However, our observations of brood‐rearing parents were consistent with previous estimates of a dependent period of 5–6 weeks (Meanley, 1969).

During a period of unusually high water levels in natural marshes, king rails with broods remained within the impoundments where water level remained constant, but returned to natural marsh when water levels receded to normal levels. This underscores the selection of habitats with shallow water during the brood‐rearing period. As broods moved an average maximum distance of nearly 600 m from their nests, we urge caution against the assumption that brood locations are representative of nesting areas and vice versa. Additionally, because rail broods may converge on favorable habitat, estimating brood survival rates without identifiable parents may result in inaccuracies.

## 5 Conclusions

Variation in seasonal habitat requirements highlights the importance of year‐round investigations into the king rail and of other declining species. Seasonal variation in home range size and unexpected rates of movement, especially during the brood‐rearing period, reveal greater per capita space requirements than previously reported. Marshes with high interspersion vary in water depth providing both optimal foraging opportunities and emergent vegetation for nesting and cover throughout the year for wading birds. Although use by rails of wetlands impounded for agricultural and wildlife management purposes has been previously documented, an unexpected finding of this study was the frequency of movement between habitats with dynamic versus stable water levels at critical life stages. While hydrologic variability can increase abundance of emergent vegetation (Galat et al., 1998), habitat heterogeneity (Rehm & Baldassarre, 2007), and macroinvertebrate diversity (Voigts, 1976), areas with stable water levels were important especially for brood‐rearing king rails.

## Conflict of Interest

None declared.
